# Adverse Effects of Metformin From Diabetes to COVID-19, Cancer, Neurodegenerative Diseases, and Aging: Is VDAC1 a Common Target?

**DOI:** 10.3389/fphys.2021.730048

**Published:** 2021-10-04

**Authors:** Varda Shoshan-Barmatz, Uttpal Anand, Edna Nahon-Crystal, Marta Di Carlo, Anna Shteinfer-Kuzmine

**Affiliations:** ^1^Department of Life Sciences, Ben-Gurion University of the Negev, Beersheba, Israel; ^2^National Institute for Biotechnology in the Negev, Ben-Gurion University of the Negev, Beersheba, Israel; ^3^Achva Academic College, Arugot, Israel; ^4^Institute for Biomedical Research and Innovation, National Research Council, Palermo, Italy

**Keywords:** apoptosis, cancer, metabolism, metformin, hexokinase, COVID-19, mitochondria, VDAC

## Abstract

Metformin has been used for treating diabetes mellitus since the late 1950s. In addition to its antihyperglycemic activity, it was shown to be a potential drug candidate for treating a range of other diseases that include various cancers, cardiovascular diseases, diabetic kidney disease, neurodegenerative diseases, renal diseases, obesity, inflammation, COVID-19 in diabetic patients, and aging. In this review, we focus on the important aspects of mitochondrial dysfunction in energy metabolism and cell death with their gatekeeper VDAC1 (voltage-dependent anion channel 1) as a possible metformin target, and summarize metformin’s effects in several diseases and gut microbiota. We question how the same drug can act on diseases with opposite characteristics, such as increasing apoptotic cell death in cancer, while inhibiting it in neurodegenerative diseases. Interestingly, metformin’s adverse effects in many diseases all show VDAC1 involvement, suggesting that it is a common factor in metformin-affecting diseases. The findings that metformin has an opposite effect on various diseases are consistent with the fact that VDAC1 controls cell life and death, supporting the idea that it is a target for metformin.

## Overview: Metformin’s Molecular and Cellular Aspects, Proposed Targets, and Therapeutic Mode of Action

Metformin is a biguanide derivative [3-(diaminomethylidene)-1,1-dimethylguanidine] that was first extracted from the flowers of goat’s rue, the French lilac (Galega officinalis) (Bailey, 2017). Here, we present its reported effects, in addition to type 2 diabetes mellitus (T2DM) (Viollet et al., 2012), on several diseases such as cancer and cardiovascular and neurodegenerative diseases (Ghatak et al., 2011; Mazza et al., 2012; Rizos and Elisaf, 2013; Foretz et al., 2014; Kasznicki et al., 2014; Scheen et al., 2015; Hitchings et al., 2016; Novelle et al., 2016; Lv and Guo, 2020; Dardano and Del Prato, 2021). The complex and heterogeneous molecular basis of these diseases suggests that many biological signaling pathways are influenced by metformin; therefore, it is very difficult to pin down its underlying mechanism(s) of action. It is proposed that it acts on metabolism (Da Silva et al., 2010), which is tightly linked to the cell signaling pathways involved in proliferation and survival, with their dysregulation associated with various diseases. Metformin acts via multiple mechanisms/signaling pathways including AMP-activated kinase (AMPK) signaling, the mammalian target of rapamycin (mTOR) (Pernicova and Korbonits, 2014; Howell et al., 2017), and inflammatory, mitochondrial (Owen et al., 2000; Foretz et al., 2014; Luengo et al., 2014), and insulin signaling, as well as cell death signaling whose dysregulation is associated with some diseases (Viollet et al., 2012). Metformin increased the ratio of AMP/ATP and suppressed mitochondrial respiratory chain complex I, resulting in increased AMPK signaling, and reduced glucagon signaling (Pernicova and Korbonits, 2014*).* Metformin downregulates oxidative phosphorylation genes, AKT and p38, and type I interferon response pathways (interleukin 1β and interferon γ) (Titov et al., 2019), inhibits mTOR (Kalender et al., 2010; Howell et al., 2017), stimulates the blood cellular landscape, and increases reactive oxygen species (ROS) production (Mogavero et al., 2017). Moreover, metformin treatment has been associated with various classifications of age-related cognitive decline, showing mixed results with both positive and negative findings (Campbell et al., 2018).

A special issue devoted to “Metformin: Beyond Diabetes” has recently been published (Bost et al., 2019). Here, we focused on the functions of the mitochondria and their governor protein VDAC1 in the effects of metformin. Mitochondria are responsible for produce energy and perform other functions associated with essential metabolism and cell signaling. Mitochondrial dysfunction is present in many diseases from T2DM to cancer, cardiovascular diseases, obesity, renal diseases, and all neurodegenerative diseases (Sorrentino et al., 2018). Moreover, we have introduced VDAC1 as a protein that possibly mediates the multiple effects of metformin. It is overexpressed in several diseases, and its overexpression is induced by apoptosis inducers. Accumulated data showed that VDAC1 overexpression is common in many diseases (T2DM, cancer, Alzheimer’s’ disease, Parkinson’s disease, cardiovascular diseases, and more) that are affected by metformin-affecting diseases (Table 1). The relationship between VDAC1 and the reported diverse effects of metformin and the major proposed metformin mechanisms of action are presented here.

**TABLE 1:** Voltage-dependent anion channel 1 overexpression is a common factor in metformin-affecting diseases.

Diseases	VDAC1 state	Function	Ref.	Metformin association	Ref.
Type 2 diabetes (T2DM)	Overexpressed	Impairs generation of cellular ATP and induced apoptosis	Ahmed et al., 2010; Gong et al., 2012; Sasaki et al., 2012; Zhang E. et al., 2019	Improves glucose tolerance	Maruthur et al., 2016; Palmer et al., 2016; Sanchez-Rangel and Inzucchi, 2017
Cancer	Overexpressed	Increases cancer cell metabolic activity	Abu-Hamad et al., 2006; Koren et al., 2010; Arif et al., 2014; Shoshan-Barmatz et al., 2015; Arif et al., 2017; Shoshan-Barmatz et al., 2017a; Pittala et al., 2018	Anti-cancer activity	Chen et al., 2017; Andrzejewski et al., 2018; Biondani and Peyron, 2018; Xie et al., 2020
Alzheimer’s disease (AD)	Overexpressed	Neuronal cell death	Perez-Gracia et al., 2008; Cuadrado-Tejedor et al., 2011; Manczak and Reddy, 2012	Neuroprotective	Qiu and Folstein, 2006; Hsu et al., 2011; Rotermund et al., 2018
Parkinson’s disease (PD)	Interaction with alpha-synuclein	Regulates VDAC1 conductance and VDAC1-mediated Ca^2+^ transport	Rostovtseva et al., 2015; Rosencrans et al., 2021	Reverses certain PD phenotypes	Bayliss et al., 2016; Lu et al., 2016; Ryu et al., 2018
Epilepsy	Increased expression	Apoptosis, alerts energy charge	Jiang et al., 2007	Decreases seizure frequency and duration, stops seizures	Zhao et al., 2014; Yang et al., 2017; Nandini et al., 2019
Depression/Bipolar disease	Upregulation of VDAC and TSPO	TSPO-VDAC complex down-regulates mitophagy proteins and NLRP3 inflammasome activation	Nahon et al., 2005; Scaini et al., 2019	Anti-depressant	Guo et al., 2014
Cardiovascular diseases (CVDs)	Overexpressed	Cardiomyocyte cell death	Lim et al., 2001; Schwertz et al., 2007; Liao et al., 2015; Tong et al., 2017; Jiang et al., 2018; Tian et al., 2019; Yang et al., 2019; Klapper-Goldstein et al., 2020	Reduces risk of CVDs among patients with T2DM	Norwood et al., 2013; Griffin et al., 2017; Rena and Lang, 2018; Mohan et al., 2019
Non-alcoholic fatty liver disease (NAFLD)	Overexpressed	Mediates transport of fatty acids across the OMM	Lee et al., 2011; Tonazzi et al., 2015; Pittala et al., 2019	Attenuates the onset of NAFLD	Koren et al., 2010; Brandt et al., 2019
Inflammatory bowel disease (IBD) and gut microbiota composition	Overexpressed	Mediates apoptosis, and inflammation	Verma et al., 2021	Affects IBD and intestinal microbiota and is a barrier in small intestine	Brandt et al., 2019; Ouyang et al., 2020; Tseng, 2021
Rheumatoid arthritis (RA)	Increased VDAC1 oligomerization	Induces cardiac cell death and functional impairment in RA	Zeng et al., 2018	Improves the pathogenesis of RA	Matsuoka et al., 2020
COVID-19	Overexpressed	Induction of apoptosis	Thompson et al., 2020	Decreases risk of death in T2DM affected by COVID-19	Chen L. et al., 2020; Luo et al., 2020; Scheen, 2020; Bramante et al., 2021

## Voltage-Dependent Anion Channel: Isoforms, Mitochondria Function, and Overexpression

Mitochondria play a fundamental role in metabolism, not only by producing the main energy for cellular functions, but they also play a crucial role in almost all aspects of cell biology and regulate cellular homeostasis, metabolism, innate immunity, cell death (apoptosis, necroptosis, pyroptosis ferroptosis, autophagy, necrosis), epigenetics, and more (Wallace, 2005; Mcbride et al., 2006; Murphy and Hartley, 2018). Because mitochondria metabolism dysregulation is associated with several severe diseases, mitochondria are a potential target for therapeutic intervention (Schirrmacher, 2020).

Mitochondria contain about 1,000 different proteins with different functions that depend on the exchange of metabolites and ions between the cytosol and mitochondria. Therefore, metabolites must be transported across both the outer mitochondrial membrane (OMM) and the inner mitochondrial membrane (IMM). The voltage-dependent anion channel 1 (VDAC1) allows the transfer of metabolites across the OMM, while the IMM is equipped with many transporters, the carrier proteins, each of which is responsible for transporting specific metabolites across the IMM (Shoshan-Barmatz et al., 2010, 2015; Colombini, 2016; Shoshan-Barmatz et al., 2017a,b; De Pinto, 2021).

**FIGURE 1:**
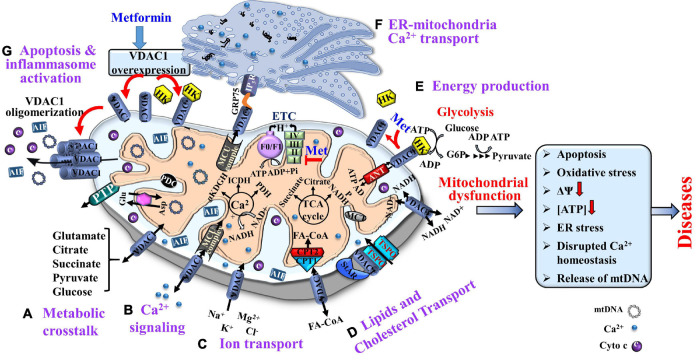
Voltage-dependent anion channel 1, a multi-functional protein, controlling cell and mitochondria functions. VDAC1 functions include: **(A)** Controlling metabolic crosstalk between mitochondria and the rest of the cell; **(B)** Acting as a Ca^2+^ transporter in and out of the intermembrane space (IMS); **(C)** Mediating ion transport including Ca^2+^ that was transported by the MUC complex into the matrix, where Ca^2+^ regulates energy production via activation of the TCA cycle enzymes: pyruvate dehydrogenase (PDH), isocitrate dehydrogenase (ICDH), and α-ketoglutarate dehydrogenase (α-KGDH); **(D)** Mediating lipid transport such as of acyl-CoAs across the OMM to the IMS, to be converted into acylcarnitine by CPT1a for further processing by β-oxidation and the transported cholesterol; **(E)** Mediating cellular energy production by transporting ATP/ADP and NAD^+^/NADH from the cytosol to the IMS, and regulating glycolysis via association with HK. Metformin induces HK detachment; **(F)**. Involvement in structural and functional association of the mitochondrion with the ER; and **(G)** Participation in apoptosis via its oligomerization, allowing cytochrome *c* release, and apoptotic cell death and mtDNA release that triggers inflammosome activation. Metformin induced VDAC1 overexpression, VDAC1 oligomerization, and apoptosis. As HK is acting as an anti-apoptotic protein, its detachment by metformin further enhances apoptosis. The TCA cycle, electron transport chain (ETC), ATP synthase (F_*o*_F_1__)_, and key ER-mitochondria association proteins are indicated. It is proposed that metformin may affect pathological conations that are associated with dysfunction of mitochondria activities.

Thus, VDAC1, as a multi-functional protein, is a key regulator of mitochondrial function serving as a mitochondrial gatekeeper. It controls the metabolic and energetic crosstalk between the mitochondria and the rest of the cell, and it is also one of the key proteins in mitochondria-mediated apoptosis (Shoshan-Barmatz et al., 2010, 2015; Shoshan-Barmatz and Ben-Hail, 2012; Magri et al., 2018; De Pinto, 2021; Figure 1).

In mammals, three isoforms of VDAC (VDAC1, VDAC2, and VDAC3) have been identified and shown to share many structural and functional properties (De Pinto et al., 2010; Raghavan et al., 2012; Zeth and Zachariae, 2018; Messina et al., 2012). The three isoforms are expressed in most tissue types, with VDAC1 expression being higher in most, but not all tissues than that of VDAC2 and VDAC3. VDAC1 is also the most abundant and best studied isoform (De Pinto et al., 2010; Messina et al., 2012; Raghavan et al., 2012), and VDAC2 was reported as a pro-apoptotic protein, interacting with Bax (Roy et al., 2009), yet its effect in apoptosis is controversial (Maurya and Mahalakshmi, 2016), expressed mainly in cancer, but not in the brain.

Voltage-dependent anion channel 1 is composed of 19 transmembrane β-strands connected by flexible loops, forming a β-barrel, and a 26-residue-long N-terminal region that lies inside the pore (Bayrhuber et al., 2008; Hiller et al., 2008; Ujwal et al., 2008). However, the N-terminus domain can be translocated from the internal pore to the channel surface (Geula et al., 2012a), and it can interact with hexokinase (HK) (Azoulay-Zohar et al., 2004; Zaid et al., 2005; Abu-Hamad et al., 2008; Shoshan-Barmatz et al., 2008a, 2010; Neumann et al., 2010), Aβ (Thinnes, 2011; Smilansky et al., 2015), and other proteins such as Bcl-2 and Bcl-xL (Shimizu et al., 1999, 2000; Malia and Wagner, 2007; Abu-Hamad et al., 2009; Arbel and Shoshan-Barmatz, 2010; Shoshan-Barmatz et al., 2010; Arbel et al., 2012).

Purified and membrane-embedded VDAC1 can assemble into dimers, trimers, tetramers, hexamers, and higher-order complexes (Zalk et al., 2005; Shoshan-Barmatz et al., 2008b, 2010, 2013, 2015, 2017a,b; Zeth et al., 2008; Keinan et al., 2010; Betaneli et al., 2012; Geula et al., 2012a; Shoshan-Barmatz and Golan, 2012; Shoshan-Barmatz and Mizrachi, 2012; Boulbrima et al., 2016). Contact sites between VDAC1 molecules in dimers and higher oligomers have also been identified (Geula et al., 2012b).

The positioning of VDAC1 at the OMM also allows its interaction with proteins involved in the integration of mitochondrial functions with other cellular activities. Indeed, VDAC1 is considered a hub protein, as it interacts with over 100 proteins (Rostovtseva and Bezrukov, 2008; Shoshan-Barmatz et al., 2017a,b; Kanwar et al., 2020).

It functions as a docking site for diverse mitochondrial, cytosolic, nuclear and ER proteins that together mediate and/or regulate metabolic, apoptotic, and other processes in normal and diseased cells. The VDAC1 interactome includes proteins that are involved in signal transduction anti-oxidation, metabolism, apoptosis, DNA- and RNA-linked proteins, and more (Caterino et al., 2017; Shoshan-Barmatz et al., 2017a,b).

VDAC1 interacts with proteins involved in energy homeostasis such as adenine nucleotide translocase (ANT), tubulin, glycogen synthase kinase (GSK3), creatine kinase, and hexokinase (HK), and it interacts with proteins that regulate apoptosis such as Bax, Bcl-2, and Bcl-xL, and in HK functions as an anti-apoptotic protein (Shoshan-Barmatz et al., 2010; Shoshan-Barmatz and Mizrachi, 2012; Shoshan-Barmatz et al., 2017a,b). Thus, VDAC1 appears to be a convergence point for a variety of cell survival and death signals, mediated through its association with various ligands and proteins that link energy, redox. Thus VDAC1 signaling pathways in mitochondria and other cell compartments (Figure 1).

VDAC1 functions as a hub protein that regulates ATP production, Ca^2+^ homeostasis, and apoptosis —all crucial for proper mitochondrial function and, consequently, for normal cell physiology. Thus, alterations in VDAC1 functions are associated with mitochondrial dysfunction.

This is well demonstrated by silencing VDAC1 expression in cell lines and different cancer mouse models using specific siRNAs. We demonstrated that silencing this expression resulted in metabolic reprogramming that altered the expression of over 2,000 genes, many of which belong to mitochondria, glycolysis, and other pathways associated with metabolism. Moreover, VDAC1 silencing inhibited tumor growth, modulated the tumor microenvironment, eliminated tumor oncogenic properties (e.g., angiogenesis, stemness), and induced differentiation into normal-like cells (Arif et al., 2014, 2017, 2018, 2019a,b; Amsalem et al., 2020).

The association of VDAC1 with various diseases (Shoshan-Barmatz et al., 2020; Varughese et al., 2021) is reflected in its overexpression. VDAC1 is overexpressed in cancer (Arif et al., 2014, 2017; Shoshan-Barmatz et al., 2015; Shoshan-Barmatz et al., 2017a,b; Pittala et al., 2018), Alzheimer’s disease (AD) (Perez-Gracia et al., 2008; Cuadrado-Tejedor et al., 2011; Manczak and Reddy, 2012), T2DM (Ahmed et al., 2010; Sasaki et al., 2012; Zhang et al., 2019), autoimmune diseases such as lupus (Kim et al., 2019b), cardiovascular diseases (CVDs) (Klapper-Goldstein et al., 2020), inflammatory bowel diseases (IBDs) (Verma et al., 2021), non-alcoholic fatty liver disease (NAFLD) (Pittala et al., 2019), COVID-19 (Luo et al., 2020; Scheen, 2020; Bramante et al., 2021), and others (Table 1). As VDAC1 overexpression induces apoptotic cell death (Godbole et al., 2003; Zaid et al., 2005; Abu-Hamad et al., 2006; Ghosh et al., 2007; Weisthal et al., 2014), its overexpression in these diseases may be a common mechanism in their pathologies. It is not clear whether VDAC1 overexpression leads to the disease or if the disease state results in VDAC1 overexpression.

In post-mortem brain of patients with Down Syndrome (DS) and Alzheimer’s disease (AD), the levels of VDAC1 and VDAC2 were altered (Yoo et al., 2001). In the DS cerebellum, total VDAC1 protein was elevated, whereas VDAC2 showed no significant alterations.

In AD brains, VDAC1 was significantly decreased in the frontal cortex and thalamus. VDAC2 was significantly elevated only in the temporal cortex. However, other studies showed that, in AD, VDAC1 is overexpressed early in the disease (Fernandez-Echevarria et al., 2014).

Finally, in cancer, VDAC1 (Abu-Hamad et al., 2006; Koren et al., 2010; Arif et al., 2014, 2017; Shoshan-Barmatz et al., 2015, Shoshan-Barmatz et al., 2017a,b; Pittala et al., 2018) and VDAC3 (Jozwiak et al., 2020) are overexpressed, and shown to be essential for cancer development (siRNA). VDAC2 was found to be required, for BAX-mediate apoptosis (Chin et al., 2018).

This review focuses on the relationship between VDAC1 and the reported diverse effects of metformin. For other VDAC isoforms, no published data are available, except for a report demonstrating, by using a proteomic approach, that in metformin-treated MCF-7, VDAC2, was found to be upregulated along with the proapoptotic proteins p53, Bax, and Bad (Al-Zaidan et al., 2017). With respect to T2DM, pancreatic β-cells express both VDAC1 and VDAC2 (Ahmed et al., 2010; Zhang et al., 2019). Under glucotoxic conditions (20 mM glucose), INS-1E cells significantly overexpressed VDAC1, whereas VDAC2 levels were reduced (Ahmed et al., 2010). However, islets from T2D pancreas donors show upregulated *VDAC1* mRNA, while *VDAC2* mRNA is suppressed, compared with islets in healthy donors (Zhang et al., 2019). In addition, in T2D β cells, VDAC1 levels were decreased in endoplasmic reticulum–mitochondria contact sites (Thivolet et al., 2017). Thus, not only altered *VDAC* gene expression, but also its sub-cellular localization could lead to mitochondrial dysfunction. Thus, the involvement of VDAC2 in metformin effects can not be ruled out due to its cellular functions in apoptosis (Naghdi and Hajnoczky, 2016).

Metformin also has been reported to increase VDAC1 expression levels in NCaP cells along with increased the levels of IP3R_1_, IP3R_2_, IP3R_3_, and MCU mRNA, as well as VDAC1 protein (Loubiere et al., 2017), and in polycystic ovary syndrome (PCOS)-like rats treated with metformin. Zhang et al. (2017) revealed that treatment with metformin increased VDAC expression and decreased superoxide dismutase 1 (SOD1) in PCOS-like rats compared to control rats (Zhang et al., 2017). In addition, metformin in the presence of citral, but not in its absence, increased VDAC expression (Duan et al., 2021). Moreover, VDAC1 is overexpressed in diseases that were found to be modulated by metformin (Table 1).

## Metformin Mode of Action

**FIGURE 2:**
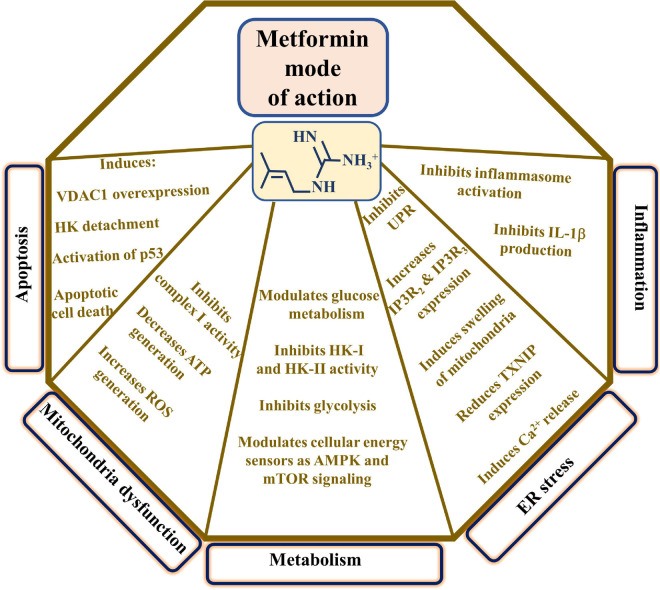
Proposed metformin mode of action. The major proposed metformin mechanisms of action such as inducing apoptosis, mitochondrial dysfunction, modulating cell metabolism, and affecting ER stress and inflammation are presented. These are reflected in the indicated metformin effects. TXNIP indicates thioredoxin-interacting protein and UPR, the unfolded protein response.

The major proposed metformin mechanisms of action include modulating cell metabolism, inducing apoptosis, mitochondrial dysfunction, ER stress, inflammation, and more (Vial et al., 2019; Figure 2). These proposed mechanisms point to the complexity of metformin action at the molecular and cellular levels, as presented below.

### Metformin Modulation of AMPK and mTOR Signaling, and Mitochondrial Functions

Metformin acts on the central cell metabolism and on several major signaling pathways including glucose metabolism and energy-sensing that involve the cellular energy sensor AMPK and mTOR signaling (Howell et al., 2017). It has been shown that metformin activates the AMPK pathway via ATM (ataxia telangiectasia mutated), LKB1 (liver kinase B1) activation, and inhibition of the mTOR pathway, leading to a reduction in protein synthesis and cell growth (Howell et al., 2017). Metformin can activate p53 by activating AMPK, thereby, inhibiting the cell cycle (Saraei et al., 2019).

AMP-activated kinase activation is required for gluconeogenesis suppression and stimulation of glucose uptake by peripheral tissues (Musi et al., 2002). However, it was recently shown that metformin inhibits hepatic gluconeogenesis in transgenic mice without AMPK or its upstream activator LKB1 (Foretz et al., 2014).

It is widely documented that metformin is one of the most potent drugs that activates AMPK (Foretz et al., 2014). Stimulating AMPK activity, affects age-related disorders including cancer, CVDs, diabetes, neurocognitive decline, and more (Wang et al., 2011; Coughlan et al., 2014). Activation of AMPK initiates the phosphorylation of tuberin and raptor (mTOR cascade proteins), leading to the rapid inhibition of mTOR pathway activity (Shaw, 2009).

At the same time, there are several cellular targets that can drive the metformin effect independently from AMPK (Viollet et al., 2012; Foretz et al., 2014). These include the electron chain complexes (ETCs) I (Owen et al., 2000), II, and IV (Drahota et al., 2014), serine-threonine liver kinase B1/AMP-activated protein kinase complex (LKB1/AMPK) (Shaw et al., 2005), adenylate cyclase (Miller et al., 2013), AMP deaminase (Ouyang et al., 2011), NADPH oxidase (Piwkowska et al., 2010), and mitochondrial glycerophosphate dehydrogenase (Madiraju et al., 2014). However, other targets are also proposed such as HK and VDAC1 (see section “Hexokinase-VDAC1 Interaction as a Metformin Target”).

Metformin affects glucose consumption, lactate production, oxidative metabolism, and ATP levels similarly to those promoted by insulin alone, suggesting that metformin modulates the key enzymes involved in glycolysis regulation such as HK and phosphofructokinase (PFK) (Da Silva et al., 2010).

Accumulated evidence suggests the involvement of mitochondria in metformin activities (Vial et al., 2019), and raises the question of exactly how metformin enters the mitochondria. Metformin distribution and cell penetration are mediated by tissue-specific transporters counting plasma membrane monoamine transporter (PMAT) in the intestine, organic cation transporter 1 (OCT1) in the liver, and both organic cation transporter 2 (OCT2) and multidrug and toxin extruder (MATE)1/2 in the kidneys (Gormsen et al., 2016). It should be noted that exactly how metformin enters the mitochondria is unclear (Fontaine, 2014). The intra-mitochondrial accumulation of phenformin, another biguanide, has been shown to involve the mitochondrial organic cation/carnitine transporter 1 (OCTN1) (Shitara et al., 2013). Metformin affecting mitochondrial function via modulation of the multifunctional OMM protein VDAC1 modulation requires no metformin transport into the mitochondria.

One of the proposed metformin targets is the mitochondrial respiratory chain protein complex-I (Owen et al., 2000; Foretz et al., 2014; Luengo et al., 2014), but it is not clear if metformin inhibits complex-I by direct interaction (Fontaine, 2014). In isolated mitochondria, very high concentrations of metformin (20–100 mM) inhibit complex-I activity, while micromolar concentrations are required for its inhibition in various cell types (El-Mir et al., 2000) or *in vivo* in skeletal muscle from healthy and diabetic rats (Wessels et al., 2014). It should be noted that clinically relevant metformin concentrations are <100 μM.

Several explanations have been proposed for this discrepancy between the metformin concentration required for complex-I inhibition in the *in vitro* and *ex vivo* experiments and the clinically relevant concentrations (He and Wondisford, 2015), including that the positive charge of metformin slows it accumulation within the matrix due to the transmembrane electrochemical potential (ΔΨ) (Bridges et al., 2014).

Metformin inhibiting complex-I activity reduces ATP production and elevates the levels of AMP and ADP. The increase in the AMP/ATP ratio with increased AMP leads to inhibition of gluconeogenesis and activation of AMPK (Miller et al., 2013; Foretz et al., 2014; Pernicova and Korbonits, 2014).

### Hexokinase-VDAC1 Interaction as a Metformin Target

The first step of glycolysis is catalyzed by HK, with the isoforms HK-I or HK-II known to bind to the OMM through VDAC1 (Azoulay-Zohar et al., 2004; Zaid et al., 2005; Abu-Hamad et al., 2008; Shoshan-Barmatz et al., 2008a, 2010; Neumann et al., 2010; Shoshan-Barmatz et al., 2017a,b). This has a metabolic benefit as phosphorylation of glucose by VDAC1-bound HK is coupled to the mitochondrial-produced ATP with ATP channeling enhancing glycolysis. The binding of HK to VDAC1 has another important aspect in inhibiting cytochrome *c* (Cyto c) release and, subsequently, apoptosis occurring in cells expressing native, but not E-72Q-mutated VDAC1 (Zaid et al., 2005; Abu-Hamad et al., 2008; Arzoine et al., 2009). Hence, HK by binding to VDAC1, provides the cell with both a metabolic benefit and apoptosis suppression.

Metformin has been shown to directly inhibit the enzymatic activity of HK-I and HK-II through an allosteric modification of HK structure, leading to the inhibition of glucose-6-phosphate (G-6-P) production, thereby, inhibiting glycolysis (Marini et al., 2013; Salani et al., 2013; Picone et al., 2016). Also, metformin induces the detachment of HK-II from its binding site in the OMM (Salani et al., 2013, 2014). HK-I or HK-II detachment from the mitochondria has been shown to activate apoptosis (Azoulay-Zohar et al., 2004; Zaid et al., 2005; Abu-Hamad et al., 2008; Shoshan-Barmatz et al., 2008a, 2010). Thus, metformin detachment of HK-I/HK-II is expected to result in apoptotic cell death. Indeed, metformin acting through HK and VDAC1 not only impairs metabolism, but also induces mitochondrial dysfunction and cell death (Marini et al., 2013; Salani et al., 2013). This may explain the pro-apoptotic effect of metformin on cancer cells that overexpress HK-I/HK-II (Smith, 2000).

Detaching HK from VDAC1 has also been shown to impair glutamate transporter-mediated glutamate uptake (Jackson et al., 2015). Thus, it is expected to impair the uptake of excitatory neurotransmitter glutamate, affecting synaptic activity.

Collectively, the above strongly suggests that metformin’s mode of action involves the mitochondria, as inhibition of complex I, glycerophosphate dehydrogenase, and HK can affect the NAD/NADH ratio and ATP production. Also, as emphasized above, the HK–VDAC1 complex is critical in metabolism and apoptosis, and in detaching HK from VDAC1, leading to impairment of mitochondrial activity and apoptosis induction. Moreover, metformin increases VDAC1 expression levels, shifting the equilibrium from monomeric to oligomeric VDAC1, thereby, leading to apoptotic cell death. Detachment of HK from VDAC1 and induction of VDAC1-associated cell death can explain metformin’s anti-cancer effect via the induction of apoptosis.

The mechanisms underlying metformin’s protective effects in several diseases, and the link between metformin, HK and VDAC1 are presented below (section “Cancer, Metformin, VDAC1, and HK”). Among the proposed metformin neuroprotection activity is its inhibition of the lipid phosphatase Src homology 2 domain, containing inositol-5-phosphatase 2 (SHIP2), which when elevated, reduces Akt (protein kinase B) activity (Hori et al., 2002). Metformin, by inhibiting SHIP2 activity, stimulates Akt activity, and thus, the phosphorylation of HK by Akt, which was shown to increase its binding to VDAC (Roberts et al., 2013), thereby, protecting against apoptosis.

### Metformin Modulating Apoptosis: Mitochondria, VDAC1, and HK as Key Factors

Along with regulating cellular energy and metabolism, VDAC1 is involved in mitochondria-mediated apoptosis, participating in the release of apoptotic proteins, and interacting with the anti-apoptotic proteins, Bcl_2_ and Bcl-xL, and HK, overexpressed in cancers (Figure 1G).

Apoptotic signals change the mitochondrial membrane permeability, allowing the release of apoptogenic proteins such as Cyto *c*, apoptosis-inducing factor (AIF), and SMAC/Diablo from the intermembrane space (IMS) into the cytosol (Kroemer et al., 2007; Shoshan-Barmatz et al., 2015). These proteins participate in complex processes, leading to the activation of proteases and nucleases, thereby to degradation of proteins and DNA, and cell death. Several hypotheses regarding the mechanism of mitochondria-mediated apoptosis have been proposed (Garrido et al., 2006). Our and others’ studies demonstrated that upon apoptosis induction by various reagents such as chemotherapy drugs, arbutin, prednisolone, cisplatin, viral proteins, elevated cytosolic Ca^2+^, or UV irradiation, VDAC1 expression levels were increased (Shoshan-Barmatz et al., 2020). The overexpressed VDAC1 leads to its oligomerization to form a large pore, allowing the release of mitochondrial pro-apoptotic proteins (Zalk et al., 2005; Shoshan-Barmatz et al., 2008b, 2013; Ujwal et al., 2009; Keinan et al., 2010; Huang et al., 2015; Ben-Hail et al., 2016). We further demonstrated that VDAC1 oligomerization is a dynamic process, and that it is a general mechanism common to numerous apoptotic stimuli, acting via different initiating cascades (Zalk et al., 2005; Shoshan-Barmatz et al., 2008b, 2013; Keinan et al., 2010, 2013; Weisthal et al., 2014; Huang et al., 2015; Ben-Hail and Shoshan-Barmatz, 2016; Ben-Hail et al., 2016). Moreover, recently, we identified VDAC1-interacting molecules such as diphenylamine-2-carboxylate (DPC) (Ben-Hail and Shoshan-Barmatz, 2016) and new molecules developed in our lab such as VBIT-4 and VBIT-12 that were found to prevent VDAC1 oligomerization and subsequent apoptosis. Furthermore, cyathin-R, a cyathane-type diterpenoid, was found to induce apoptosis in Bax/Bak-depleted cells, but not when VDAC1 that was inhibited by DPC was depleted (Huang et al., 2015).

Based on these results we proposed a novel model in which VDAC1 exists in a dynamic equilibrium between monomeric and oligomeric states, with apoptosis inducers shifting the equilibrium toward oligomers, forming a large channel that enables Cyto c release, leading to cell death (Zalk et al., 2005; Shoshan-Barmatz et al., 2008b, 2013; Keinan et al., 2010; Kasznicki et al., 2014; Huang et al., 2015; Ben-Hail et al., 2016). Furthermore, a correlation between drug effectiveness in apoptosis induction and VDAC1 expression levels has been reported (Castagna et al., 2004; Lai et al., 2006; Tajeddine et al., 2008). Moreover, not only apoptosis inducers, but also stress and pathological conditions can induce VDAC1 overexpression and, thus, trigger apoptosis (Keinan et al., 2013; Weisthal et al., 2014; Shoshan-Barmatz et al., 2020).

Metformin-induced apoptosis (Ben Sahra et al., 2010a; Malki and Youssef, 2011; Sancho et al., 2015) can be mediated via inducing VDAC1 overexpression and its oligomerization. Treatment of mice for 3 months with metformin increased the expression of VDAC1 in the cortex, but not in the hippocampus (Wijesekara et al., 2017). Moreover, in the cortical region, plasmalemmal VDAC1 (pl-VDAC1) was found as oligomers in areas where metformin induced Aβ-aggregate accumulation, and apoptotic neurons were observed (Wijesekara et al., 2017). In addition, metformin increased VDAC1 expression levels in NCaP cells (Loubiere et al., 2017) and in polycystic ovary syndrome-like rats (Zhang et al., 2017), and in the presence of citral (Duan et al., 2021).

Considering the pro-apoptotic effects of VDAC1 overexpression leading to its oligomerization, and subsequently to apoptotic cell death, we suggest that metformin, as do other apoptosis inducers and stress conditions, induces apoptosis (Ben Sahra et al., 2010a; Malki and Youssef, 2011; Sancho et al., 2015) via triggering VDAC1 overexpression and increasing the expression levels of p53, Bax, and Bad, while reducing the expression levels of Akt, Bcl-2, and Mdm2 (Malki and Youssef, 2011).

Previously, it was demonstrated that apoptosis induced by various reagents disrupted intracellular Ca^2+^ ([Ca^2+^]i) homeostasis (Keinan et al., 2013; Weisthal et al., 2014). Moreover, it has been shown that pro-apoptotic agents inducing upregulation of VDAC1 expression levels are Ca^2+^-dependent (Keinan et al., 2013; Weisthal et al., 2014; Shoshan-Barmatz et al., 2015). Metformin has been shown to induce ER stress and Ca^2+^ released from the ER and, subsequently, its uptake by the mitochondria, leading to mitochondrial swelling (Loubiere et al., 2017). Interestingly, metformin significantly increased the levels of mRNA encoding for IP_3_R_2_ and IP_3_R_3_ (Loubiere et al., 2017). Thus, the increase in cytosolic Ca^2+^ may be responsible for VDAC1 overexpression, as found for other inducers of this overexpression (Keinan et al., 2013; Weisthal et al., 2014; Shoshan-Barmatz et al., 2015).

Recently, we showed that metformin interacted with purified VDAC1, and inhibited the channel conductance of bilayer-reconstituted VDAC1 (Zhang et al., 2019). The direct interaction of metformin with VDAC1 may modulate VDAC1 activity, thereby, mitochondrial functions. This metformin-VDAC1 interaction is currently subjected to further studies. This together with metformin inducing VDAC1 overexpression and apoptotic cell death, may suggest that metformin-apoptosis induction involves VDAC1.

## Metformin’s Multiple Effects on Various Diseases

**FIGURE 3:**
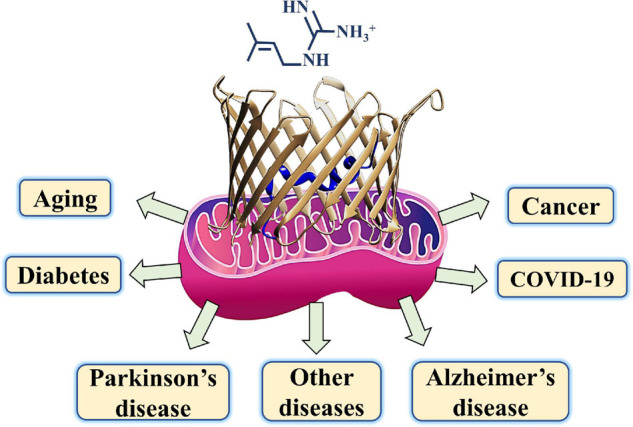
A schematic presentation of metformin targeting diseases such as diabetes, COVID-19, cancer, neurodegenerative diseases, and aging with mitochondria and their gatekeeper VDAC1 proposed as a common target.

Metformin, besides being the first-line medication used to treat T2DM, was shown to be a potential drug candidate to treat several other diseases including various cancers, cardiovascular diseases, diabetic kidney disease, neurodegenerative diseases, renal diseases, obesity, inflammation, COVID-19 in diabetic patients, and aging (Figure 3; Reina and De Pinto, 2017; Magri et al., 2018; Shoshan-Barmatz et al., 2020).

### Diabetes, Mitochondria, VDAC1, and Metformin

Diabetes mellitus (DM) is a group of chronic metabolic disorders characterized by hyperglycemia that ultimately leads to damage of different body systems (American Diabetes, 2014). It is the ninth major cause of mortality worldwide (Zheng et al., 2018), exerting a global public health threat. Patients with untreated DM or prolonged hyperglycemia may suffer from polyuria, polydipsia, polyphagia, weight loss, and blurred vision (Galtier, 2010). DM is growing at epidemic proportions, becoming increasingly prevalent in all countries. It is estimated to increase to 700 million cases by the year 2045 (English and Lenters-Westra, 2018; IDF, 2019). It was also reported that in 2014, there were 422 million people who had diabetes (NCD Risk Factor Collaboration., 2016), and 5.1 million deaths among people between 20 and 79 years old were attributed to it in 2013 (Zimmet et al., 2016). Type 2 diabetes mellitus (T2DM) is more prevalent than type 1 (T1DM) and in adults, 90–95% of patients with diabetes have T2DM.

Type 2 diabetes mellitus management consists mainly of drugs that reduce insulin resistance and glucose uptake in the intestine, as well as reduce gluconeogenesis in the liver, and drugs that increase glucose excretion through the urine. These drugs include metformin, sulfonylureas, and SGLT2 (sodium-glucose transporter protein 2) inhibitors (Tan et al., 2019). The use of metformin as a therapeutic agent began in France in 1957, and was approved for use in Canada in 1972, and in the United States by the Food and Drug Administration (FDA) in 1994 for use by those with non-insulin-dependent T2DM. Today, metformin is the first-line, leading oral antidiabetic drug prescribed for the treatment of T2DM (Pernicova and Korbonits, 2014; Hotta, 2019) either alone or in combination with thiazolidinediones, sulfonylureas, or other hypoglycemic agents (Maruthur et al., 2016).

Metformin, as an anti-hyperglycemic agent, improves glucose tolerance in patients with T2DM by lowering both basal and postprandial plasma glucose (Maruthur et al., 2016; Palmer et al., 2016; Sanchez-Rangel and Inzucchi, 2017). It increases glucose uptake and utilization by intestinal cells and lactic acid formation in these and liver cells (Mccreight et al., 2016). It reduces liver glucose production, slows glucose transfer to the blood, increases glucose utilization by muscle cells (in anaerobic glycolysis, due to the suppression of mitochondrial function and aerobic respiration in these cells), lowers insulin resistance, and increases incretin activity, and especially glucagon-like peptide 1 (GLP-1), which contributes to raising insulin levels and lowering blood glucagon levels. In addition to the effects on glucose level, metformin also contributes to the suppression of fatty acid synthesis and gluconeogenesis, and the removal of insulin sensitivity reduces blood levels of LDL cholesterol and triglycerides (Viollet et al., 2012).

The proposed metformin mode of action in regulating blood glucose level is not completely understood, and multiple potential mechanisms have been proposed. It enters liver cells mainly through OCT1, and suppresses the mitochondrial respiratory chain (complex-I). It also reduces ATP production and increases AMP levels activating AMPK; inhibits glucagon-induced elevation of cAMP with reduced activation of protein kinase A (PKA); and decreases gluconeogenesis (liver glucose production) (Foretz et al., 2014; Madiraju et al., 2014). As a result, glucose depletion in the cell increases, and the cell reduces glucose formation and increases the amount of glucose transferred from the blood.

Mitochondria have been connected to the pathophysiology of diabetes with changes in their quality, quantity, and function reported to occur in diabetics (Sivitz and Yorek, 2010).

Recently, we found that VDAC1 expression levels were increased in islets from T2DM and non-diabetic organ donors under glucotoxic conditions (Zhang et al., 2019). The overexpressed VDAC1 is mistargeted to the plasma membrane of the insulin-secreting β cells, resulting in a loss of ATP, and thereby no insulin secretion occurs. Moreover, VDAC1 antibodies, as well as metformin, and specific VDAC1-interacting molecule VBIT-4, restore the impaired generation of ATP and glucose-stimulated insulin secretion in T2DM islets (Zhang et al., 2019). Furthermore, treatment of db/db mice with VBIT-4 prevents hyperglycemia, and maintains normal glucose tolerance and physiological regulation of insulin secretion (Zhang E. et al., 2019). These metformin effects are not mediated via activation of AMP kinase (Foretz et al., 2014) or through antioxidant effects such as an AMPK inhibitor (MRT199665), nor do the antioxidants N-Acetyl cysteine influence metformin’s effects.

These findings suggest that VDAC1 is a diabetes executer protein that can be targeted by its interacting molecules, as indicated above (Zhang E. et al., 2019). Moreover, high glucose enhances VDAC1 expression levels by elevating the expression of SREBP1 and SREBP2, the transcription factors of VDAC1 (Zhang E. et al., 2019).

Several recent studies have identified bacterial effectors of metformin therapy (Pryor et al., 2019), Metformin signatures in the human gut microbiome of T2DM were demonstrated using 784 available human gut metagenomes, and proposed mechanisms contributing to the beneficial effects of the drug on the host’s metabolism (Forslund et al., 2015). Metformin-induced changes in T2DM patients are expressed by the significant decrease in *Verrucomicrobia* and *Firmicutes* and an increase in *Actinobacteria* and *Bacteroidetes* (Zhang et al., 2015; De La Cuesta-Zuluaga et al., 2017; Nakajima et al., 2020). Furthermore, *Escherichia*, *Streptococcus*, *Subdoligranulum*, *Clostridium*, *Bacteroides*, and *Collinsella* were the genus-level bacteria that increased, whereas *Ruminococcus* and *Faecalibacterium* bacteria decreased (Rosario et al., 2018; Nakajima et al., 2020). For example, when metformin was given to healthy young men, their *Bilophila wadsworthia* and *Escherichia/Shigella spp.* increased, whereas their *Clostridium spp.* and *Intestinibacter spp.* decreased (Bryrup et al., 2019).

In addition, metformin was found to alter upper small intestinal microbiota that impact the sodium-dependent glucose cotransporter (SGLT1) sensing glucoregulatory pathway (Baur and Birnbaum, 2014).

The link between metformin effects on gut microbiota, and VDAC1 is not clear, yet it is likely related to VDAC1 function as transporter of verity of metabolites that their levels can be affected by the microbiota and its modulation by metformin.

### Cancer, Metformin, VDAC1, and HK

Generally, diabetic patients are more expected to develop a variety of cancers (Shikata et al., 2013; Collins, 2014; Cignarelli et al., 2018; Kang et al., 2018; Scully et al., 2020). These patients are at increased risk of developing cancers such as breast, prostate, pancreatic, and non-small cell lung (NSCLC) cancer compared to non-diabetic patients (Richardson and Pollack, 2005; Pierotti et al., 2013).

In the past decades, several epidemiologic studies have linked numerous *in vitro* and *in vivo* studies, along with epidemiological, clinical, and preclinical evidence supporting the anti-cancer activity of metformin (Ben Sahra et al., 2010b; Evans et al., 2010; Jalving et al., 2010; Dowling et al., 2011; Zhang et al., 2011; Rizos and Elisaf, 2013; Kasznicki et al., 2014; Higurashi et al., 2016; Mohamed Suhaimi et al., 2017).

The molecular mechanisms associated with the anti-cancer activity of metformin are complex and include several targets and pathways (Saini and Yang, 2018). Several potential mechanisms proposed for its ability to suppress cancer *in vitro* and *in vivo* include: (a) activation of the LKB1/AMPK pathway, (b) induction of cell cycle arrest, and/or apoptosis, (c) inhibition of protein synthesis, (d) reduction in circulating insulin levels, (e) inhibition of the unfolded protein response (UPR), (f) activation of the immune system, and (g) eradication of cancer stem cells (Kourelis and Siegel, 2012).

Metformin anticancer effects include AMP-activated protein kinase activation, mTOR inactivation, mitogen-activated protein kinase 1 (MEK)/extracellular signal-regulated kinase (ERK), and phosphatidylinositol 3-kinase (PI3K)/AKT signaling pathway inhibition (Pernicova and Korbonits, 2014). It is also suggested that metformin has an anti-tumor effect by lowering insulin levels and disabling the mTOR in the cell (Del Barco et al., 2011; Foretz et al., 2014; Zhang et al., 2018).

*In vitro*, metformin exhibits a strong anti-proliferative action on cancer cell lines derived from the breast, colon, ovaries, pancreas, lung, and prostate (Andrzejewski et al., 2018), as well as in leukemia, and pancreatic and colorectal cancers (Chen et al., 2017; Biondani and Peyron, 2018; Xie et al., 2020). Metformin in combination with 5-FU strongly inhibited colorectal cancer (Wang B. Z. et al., 2019), and it was shown to suppress cancer initiation and progression in genetic mouse models (Chen et al., 2017). It was shown to selectively inhibit metastatic colorectal cancer with the KRAS (Kirsten rat sarcoma viral oncogene homolog) mutation, inhibiting cell proliferation by inactivating both RAS/ERK and AKT/mTOR signaling (Xie et al., 2020). Metformin suppresses cancer stem cells (CSCs) in the tumor, and enhances the responsiveness of glioma cells to temozolomide (Zhang et al., 2010). It has also been demonstrated that metformin can be used as a co-adjuvant, reverting the resistant-like pattern of a human glioma cell line both *in vitro* and *in vivo* (Rattan et al., 2012).

Metformin has been shown to facilitate DNA repair, which is critical for cancer prevention (Lee et al., 2016). It was proposed that it targets pancreatic CSCs, but not their differentiated non-CSCs (Sancho et al., 2015). It is further proposed that mitochondrial inhibition by metformin creates an energy crisis and induces CSC apoptosis (Sancho et al., 2015).

In prostate cancer cells, the combination of metformin and 2-deoxyglucose (2-DG) (that binds to HK) drastically reduced intracellular ATP levels through the inhibition of the mitochondrial complex 1 and glycolysis (Ben Sahra et al., 2010a). Metformin was also shown to affect the glycolytic rate by directly inhibiting HK-II activity and its interaction with the mitochondria (Salani et al., 2014). *In silico* models suggest that metformin mimics G6P (glucose 6-phosphate) features and binds to its binding site in HK (Salani et al., 2013). The HK–VDAC1 complex formation is regulated by Akt (protein kinase B) (Majewski et al., 2004) and glycogen synthase kinase 3 beta (GSK3β), while the HK–VDAC complex is disrupted by VDAC phosphorylation (Pastorino et al., 2005). Cancer cells express high levels of mitochondria-bound HK that not only enhances glycolysis, but also protects against mitochondria-mediated apoptosis via direct interaction with VDAC1 (Bryson et al., 2002; Azoulay-Zohar et al., 2004; Zaid et al., 2005; Abu-Hamad et al., 2008, 2009; Arzoine et al., 2009). Thus, metformin inhibits HK activity and induces HK detachment from the VDAC1, resulting in both inhibiting cancer cell metabolism and inducing apoptosis. It should be noted, however, that some clinical trials have failed to show a protective association between metformin and survival in colorectal cancer (CRC) patients with T2DM (Cossor et al., 2012; Mc Menamin et al., 2016). VDAC1 has been shown as a critical protein in cancer development and survival (Mazure, 2017) and many anti-cancer compounds were shown to mediates their activity via targeting VDAC1 (Reina and De Pinto, 2017; Magri et al., 2018). Metformin has been consistently shown to reduce the risk of various types of cancer including the breast, colon, ovaries, pancreas, lung, prostate, leukemia, and colorectal cancers (Chen et al., 2017; Andrzejewski et al., 2018; Biondani and Peyron, 2018; Xie et al., 2020) (see section “Cancer, Metformin, VDAC1, and HK”). Thus, it is possible that the anticancer effects of metformin may involve some common pathophysiological mechanisms (Del Barco et al., 2011). Among these the common hallmarks of cancer are reprogramming of energy metabolism and resisting cell death (Hanahan and Weinberg, 2011).

Cancer cells need excess energy and metabolites are required for cell proliferation and migration to distant organs for metastasis. Metabolic reprogramming in cancer cells is a significant pathogenic mechanism in cancer involving flexibility of the metabolic machinery. VDAC1, by regulating the metabolic and energetic functions of mitochondria, controls the fate of cancer cells. The overexpression of VDAC1 in various tumors obtained from patients, and in tumors established in mouse models, as well as in cancer cell lines (Arif et al., 2014, 2017; Shoshan-Barmatz et al., 2015, Shoshan-Barmatz et al., 2017a,b; Pittala et al., 2018), points to its significance in high energy-demanding cancer cells. Indeed, the pivotal role of VDAC1 in regulating cancer cellular energy, metabolism, and viability is reflected in the findings that downregulation of VDAC1 expression reduced cellular ATP levels, metabolite exchange between the mitochondria and cytosol cell proliferation, and tumor growth (Abu-Hamad et al., 2006; Koren et al., 2010; Shoshan-Barmatz and Golan, 2012; Arif et al., 2014, 2017).

Metformin via interacting with VDAC1 and modulating its conductance it can affect cancer cell metabolism. Metformin also blocks the Warburg effect in energy metabolism of cancer cells (Del Barco et al., 2011). Metformin is well recognized for its effects on the activation of AMPK, followed by the inhibition of mTOR (Viollet et al., 2012) and its activation is commonly observed in many types of cancer cells (Hanahan and Weinberg, 2000).

Another hallmark of cancer cells is their ability to suppress pro-apoptotic pathways and/or to activate anti-apoptotic mechanisms (Fulda, 2009; Hanahan and Weinberg, 2011) associated with drug resistance (Johnstone et al., 2002). Cancer cells overexpress anti-apoptotic proteins, such as the Bcl-2 family of proteins and HK, preventing the release of Cyto c from the mitochondria. VDAC1, by interacting with the anti-apoptotic proteins and HK, protects tumor cells from cell death (Shimizu et al., 1999, 2000; Pastorino et al., 2002, 2005; Pedersen et al., 2002; Shi et al., 2003a,b; Azoulay-Zohar et al., 2004; Zaid et al., 2005; Mathupala et al., 2006; Malia and Wagner, 2007; Abu-Hamad et al., 2008, 2009; Arzoine et al., 2009; Arbel and Shoshan-Barmatz, 2010; Shoshan-Barmatz et al., 2010; Arbel et al., 2012; Geula et al., 2012a).

In addition, overexpression of VDAC1 is induced by various apoptosis-inducing conditions such as chemotherapy drugs, UV irradiation, and viral proteins that increase the level of VDAC1 expression, with apoptosis being correlated with VDAC1 expression levels (Shoshan-Barmatz et al., 2010, 2013, 2015, 2020; Keinan et al., 2013; Shoshan-Barmatz et al., 2017a,b). Metformin’s anti-cancer effects can be mediated through induction of VDAC1 overexpression, as shown in NCaP cells (Loubiere et al., 2017) in polycystic ovary syndrome-like rats (Zhang et al., 2017) and the presence of citral in RD cells (Duan et al., 2021). VDAC1 overexpression leads to apoptosis induction, as presented in Section “Metformin Modulating Apoptosis: Mitochondria, VDAC1, and HK as Key Factors.”

Finally, apoptosis and VDAC1 overexpression, as induced by pro-apoptotic agents, are Ca^2+^-dependent (Keinan et al., 2013; Weisthal et al., 2014; Shoshan-Barmatz et al., 2015). As VDAC1 controls intracellular Ca^2+^ homeostasis, metformin disrupting calcium fluxes involving ER and mitochondria (Loubiere et al., 2017) may involve VDAC1.

Thus, the anti-cancer effects of metformin can be mediated through induction of VDAC1 overexpression and thereby apoptosis, affecting cell metabolism and/or Ca^2+^ homeostasis.

Metformin also affects cancer cells’ resistance to various drugs via modulating the activity or levels of ATP-binding cassette (ABC) transporters. The ABC family of transporters mediate the transport of a variety of compounds at the cost of ATP hydrolysis. Among the ANC transporters are the multiple drug resistant (MDR) proteins MDR1-P-glycoprotein (Pgp), MRP1 (the multidrug resistance protein 1, ABCC1), and others, which in cancer cells can cause resistance to various drugs (Borst and Elferink, 2002). Pgp, and MRP1 confer treatment resistance via the exclusion of drugs such as etoposide, daunorubicin, vinblastine, doxorubicin, and others (El-Awady et al., 2016; Joshi et al., 2016; Wijdeven et al., 2016). For example, P-gp has been shown to be overexpressed in various cancers such as in 52% of acute lymphocytic leukemia (ALL) patients, and this is correlated with reduced survival and treatment resistance (Olarte Carrillo et al., 2017).

Finally, metformin has been shown to affect cancer cells resistance to several drugs, such as by reducing the expression of MDR protein (Table 2).

**TABLE 2:** Metformin decreases cancer cell resistance to chemotherapy.

Disease	Metformin effect	References
ALL	In patients with higher ABCB1 gene expression levels, the combined use of metformin with chemotherapy is beneficial.	Ramos-Penafiel et al., 2018
Breast cancer	Metformin reduces the expression of MDR protein markers, prevents the growth of treatment-resistant breast cancer, and fosters re-sensitization.	Davies et al., 2017
Breast cancer	Metformin re-sensitized multidrug-resistant breast cancer cells (MCF7/5-FU and MDA-MB-231) to 5-fluorouracil (5-FU), Adriamycin, and paclitaxel reduced their invasive potential and reversed the epithelial-mesenchymal transition (EMT) phenotype.	Qu et al., 2014
Nasopharyngeal carcinoma (NPC)	Metformin reduced the expression of PECAM-1, which controls the expression of the multi-drug expression of resistance-associated proteins (MRPs) that contribute to cisplatin resistance of irradiated CNE-1 cells.	Sun et al., 2020
Breast cancer	In breast cancer and MCF7/DOX cells, metformin lowers Pgp activity.	Shafiei-Irannejad et al., 2018
Triple negative breast cancer (TNBC)	Metformin increases cisplatin’s anti-proliferative, anti-migratory, and anti-invasion effects in TNBC cells. Metformin also reduces the upregulation of RAD51 expression by triggering RAD51 proteasomal degradation.	Lee et al., 2019

The molecular relationship between T2DM and tumorigenesis has not yet been fully elucidated. Previous studies have suggested that there are several factors associated with patients with T2DM, that make them more likely to develop tumors. These include insulin resistance that leads to increased levels of insulin and insulin-like growth factor (I/IGF), which could bind to receptors and activate the downstream phosphatidylinositol 3-kinase (PI3K)/Akt and mitogen-activated protein kinase (MAPK) signaling pathways, leading to cell proliferation (Gallagher and Leroith, 2010; Djiogue et al., 2013). An additional factor is inflammation, suggesting that the insulin resistance characterizing T2DM may produce a large number of cytokines, including tumor necrosis factor α (TNF-α), interleukin (IL)-6, and IL-1β (Donath and Shoelson, 2011). These cytokines activate nuclear factor-κB and Janus kinase (JAK)/signal transducer and activator of transcription 3 (STAT-3) pathways, which are important signaling pathways in tumorigenesis (Neurath and Finotto, 2011).

Type 2 diabetes mellitus patients treated with metformin showed a decreased risk of developing cancer. A study encompassing 27 clinical trials (∼24,000 patients) showed that in people at early stages of colon and rectum cancer, metformin improved recurrence-free survival by 37%, and cancer-specific survival by 42%, and in early stage prostate cancer, it increased recurrence-free survival by 17% and cancer-free survival by 42%, compared with non-metformin users (Coyle et al., 2016). In head and neck cancer, diabetic patients treated with metformin had a 46% reduction in the risk of developing this cancer type compared to non-diabetic patients (Figueiredo et al., 2016). Similarly, the risk of gastric cancers in metformin users decreased by 55% compared with non-users (Tseng, 2016). Most of the studies showed that metformin inhibited cancer development, and showed no evidence of cancer stimulation (Anisimov, 2015).

### Neurodegenerative Disorders, Mitochondria, HK, VDAC1, and Metformin

Neurodegenerative disorders (NDs) include multiple sclerosis (MS), Parkinson’s disease (PD), Alzheimer’s disease (AD), Huntington’s disease (HD), epilepsy, amyotrophic lateral sclerosis (ALS), depression, and others. In 2015, about 46 million people globally were diagnosed with dementia, and > 6 million suffered from PD (Feigin et al., 2017). Currently, there is a growing need to search for a potential medication to treat neurodegenerative disorders worldwide.

A meta-analysis of 28 longitudinal studies demonstrated that people with diabetes had a 73% increased risk of developing dementia and a 56% increased risk of developing AD compared to the general population (Campbell et al., 2018). The mechanism linking diabetes and dementia is multifactorial, with evidence supporting the involvement of chronic low-grade inflammation, oxidative stress, vascular effects, increased cerebral amyloid-β peptides, hyperinsulinemia, and brain insulin resistance, among others (Craft, 2007; Ahtiluoto et al., 2010).

Metformin crosses the blood–brain barrier (BBB) rapidly and induces various therapeutic benefits in the brain such as enhanced learning capacity, and neuroprotective effects. It also boosts memory function and anti-inflammatory activities (Labuzek et al., 2010; Pintana et al., 2012; Guo et al., 2014; Shivavedi et al., 2017).

Metformin showed pharmacological neuroprotective efficacy in neurological diseases (Ryu et al., 2018; Demare et al., 2021), including AD (Ou et al., 2018), PD (Curry et al., 2018), and HD (Sanchis et al., 2019), with its potential use enhancing neuroprotection against apoptotic cell death (El-Mir et al., 2008), stimulating neurogenesis, improving spatial memory (Wang et al., 2012; Fatt et al., 2015), and prolonging the lifespan of mice (Martin-Montalvo et al., 2013). It was found that metformin and sulfonylureas treatment decreased the occurrence of T2DM dementia, and lowered the risk of PD in T2DM patients (Hsu et al., 2011; Wahlqvist et al., 2012a,b). Furthermore, metformin was suggested as a possible therapy of choice for diabetic patients with cognitive dysfunction, acting as an indication of changes in thinking and memory function (Pintana et al., 2012).

The positive effects of metformin were linked to a decrease in the opening of the mitochondrial permeability transition pore (PTP) that prevents the release of Cyto *c* and causes cell death (Guigas et al., 2004; Detaille et al., 2005; Lablanche et al., 2011).

In addition, the lipid phosphatase Src homology 2 domain, containing inositol-5-phosphatase 2 (SHIP2), is elevated in the brain of diabetic db/db mice (Hori et al., 2002). SHIP2 overexpression reduces Akt activity and enhances apoptosis (Polianskyte-Prause et al., 2019). Metformin directly binds to SHIP2 phosphatase, and in the skeletal muscles and kidneys of db/db mice, it reduces catalytic activity and restores Akt activity, preventing apoptosis (Polianskyte-Prause et al., 2019). These findings can be connected to HK, as phosphorylation of HK by Akt increases its binding to mitochondria (Roberts et al., 2013), thereby, protecting against apoptosis. These studies suggest a link between metformin and VDAC1 in the prevention of neuronal apoptosis in these diseases.

However, metformin was reported to affect the progression and severity of AD and other forms of dementia (Campbell et al., 2018), and lower cognitive function in patients with diabetes (Moore et al., 2013). Metformin-induced cortex mitochondrial dysfunction is associated with an overall increase of the risk of AD onset (Picone et al., 2016), and mitochondria-mediated cell death was linked to neuronal death witnessed in neurological disorders and associated with caspase-mediated apoptosis (Gervais et al., 1999; Li et al., 2000; Friedlander, 2003; Petrozzi et al., 2007; Mattson et al., 2008; Radi et al., 2014).

It has been shown that brains from AD patients contain high levels of nitrated VDAC1, pointing to oxidative damage from VDAC1 (Sultana et al., 2006), and feasibly affecting cell energy and metabolite homeostasis (Ferrer, 2009). Moreover high-levels of VDAC1 were demonstrated in the dystrophic neurites of Aβ deposits in the brains of post-mortem AD patients (Yoo et al., 2001; Perez-Gracia et al., 2008; Cuadrado-Tejedor et al., 2011; Manczak and Reddy, 2012), and in the thalamuses of mice with neurodegeneration in the Batten disease model (Kielar et al., 2009), and changes in thalamic VDAC protein levels were found to be related to spatial cognitive deficits in an animal model of Wernicke–Korsakoff syndrome (Bueno et al., 2015). Interestingly, it is also reported that in AD, VDAC1 levels are decreased in the frontal cortex, and VDAC2 is elevated in the temporal cortex (Rosa and Cesar, 2016).

Overexpression of VDAC1 is associated with apoptosis (Shoshan-Barmatz et al., 2015). This overexpression in various neuronal diseases is proposed to be associated with neuronal cell destruction and HK detachment, resulting in both inhibiting cell metabolism and inducing apoptosis. Thus, HK detachment from VDAC1 by metformin can explain the reported negative effects of metformin on neurodegenerative disorders.

These findings indicate that metformin possesses both pro-survival and pro-apoptotic activities in neurodegenerative diseases, but the factors mediating these opposite effects are not clear. Here, we suggest that these metformin effects are mediated via metformin interaction with HK and VDAC1, proteins that regulate cellular energetics and cell death.

#### Alzheimer’s Disease and Metformin

Alzheimer’s disease is characterized by progressive memory loss and a decline in cognitive function. The pathological hallmarks of the AD brain include neurofibrillary tangles (NFTs; composed of abnormal hyperphosphorylated tau protein) and amyloid plaques (Aβs) (Brion et al., 1985). Tau is involved in microtubule stabilization (Johnson and Stoothoff, 2004), associated with synaptic loss, and has been correlated with cognitive impairments in AD patients (Arriagada et al., 1992).

The underlying biological mechanisms leading to sporadic forms of AD have still not been defined, but these are proposed to involve mitochondrial dysfunction, cholinergic dysfunction, Aβ plaque formation, tau accumulation, inflammation, DNA damage, inflammatory response, hormone regulation, and lysosomal dysfunction (Dorszewska et al., 2016).

Obesity, metabolic syndrome, and T2DM were proposed to contribute to impaired cognitive function, increasing the risk for the development of dementia including AD (Ott et al., 1999; Arvanitakis et al., 2004). A recent meta-analysis of longitudinal studies suggests that the relative risk for AD is approximately 1.5-fold higher among persons with T2DM (Cheng et al., 2012).

It has been shown that T2DM is correlated with twice the risk of dementia (Hsu et al., 2011). Therefore, metformin has been proposed as a potential neuroprotective agent in T2DM patients as it is able to reduce the chances of AD onset (Hsu et al., 2011). The proposed mechanism for metformin inhibiting the development of dementia in patients with diabetes is by preventing hyperinsulinemia, which contributes to amyloid-β plaque formation in the brain and the onset of AD (Qiu and Folstein, 2006). Another study showed that metformin reduced tau phosphorylation in primary neuron cultures from a tau transgenic mouse (Kickstein et al., 2010).

Overexpression of VDAC1 in affected regions of AD brains (Perez-Gracia et al., 2008; Cuadrado-Tejedor et al., 2011; Manczak and Reddy, 2012) and in β-cells of T2D (Ahmed et al., 2010; Gong et al., 2012; Sasaki et al., 2012; Zhang E. et al., 2019) has been reported. As neuron loss, mainly due to apoptosis, occurs in AD brains (Colurso et al., 2003; Lezi and Swerdlow, 2012; Sabirov and Merzlyak, 2012; Silva et al., 2012; Smilansky et al., 2015) and VDAC1 overexpression induces apoptotic cell death (Godbole et al., 2003; Zaid et al., 2005; Abu-Hamad et al., 2006; Ghosh et al., 2007; Lu et al., 2007; Weisthal et al., 2014), its overexpression in AD and in T2DM may be a common mechanism in these pathologies.

Aβ triggered HK-I detachment from mitochondria, decreasing HK-I activity in cortical neurons (Saraiva et al., 2010). In addition, in the postmortem brain tissue of AD mice and patients, HK levels were decreased, while VDAC1 levels were elevated (Cuadrado-Tejedor et al., 2011). In addition, HK-I detachment from mitochondria was observed in AD models (Rossi et al., 2020). It is well demonstrated that HK binds to VDAC1 and that its dynamic association with VDAC1 (Zaid et al., 2005; Abu-Hamad et al., 2008; Pastorino and Hoek, 2008; Arzoine et al., 2009) is known to modulate the metabolic coupling between cytosol and mitochondria by regulating both glycolysis and oxidative phosphorylation.

#### Parkinson’s Disease and Metformin

Parkinson’s disease is a progressive neurodegenerative disease characterized by both motor and non-motor features, and is the second most common neurodegenerative disorder (Rizek et al., 2016). Metformin reversed certain PD phenotypes in PD mouse models through AMPK-dependent and independent pathways (Bayliss et al., 2016; Lu et al., 2016; Ryu et al., 2018). It lowered α-synuclein phosphorylation and upregulated neurotrophic factors in a 1-methyl-4-phenyl-1,2,3,6-tetrahydropyridine (MPTP) mouse model of PD (Katila et al., 2017), and prevented the loss of dopamine-producing brain cells in a model of PD (Lu et al., 2016).

Metformin mitigated neuronal damage, strengthened antioxidant activity, and increased muscle and locomotive functions in an MPTP-triggered PD mouse model (Patil et al., 2014). Similar findings were found where metformin-ameliorated MPTP induced dysfunction of dopaminergic neurons, elevated striatal dopamine output, and improved motor injuries in a mice model via microglia-overactivation-induced neuroinflammation inhibition, and enhanced AMPK-mediated autophagy (Lu et al., 2016). Metformin also suppressed AMPK-independent development of L-DOPA-induced dyskinesia and impaired glycogen synthase kinase 3β (GSK3β) activity, without affecting elevated mTOR or ERK signaling observed in a mouse model of PD (Ryu et al., 2018). Similar results showed neuroprotective effects of metformin and inhibition of degeneration of nigrostriatal dopamine in a PD mouse model (AMPK knockout) (Bayliss et al., 2016). This raises the feasibility that metformin could be a potential therapeutic agent in suppressing complications of L-DOPA-induced motor complications in PD (Freitas et al., 2017; Sportelli et al., 2020).

α-synuclein, a presynaptic neuronal protein, interacts with VDAC1 and regulates VDAC1 conductance and VDAC1-mediated Ca^2+^ transport (Rostovtseva et al., 2015; Rosencrans et al., 2021). Metformin-induced ER stress resulted in Ca^2+^ release from the ER and its uptake by the mitochondria, leading to mitochondrial alterations (Loubiere et al., 2017). Thus, α-synuclein, by inhibiting VDAC-mediated Ca^2+^ transport, can prevent metformin-mediated mitochondrial Ca^2+^ overload and the associated mitochondria dysfunction.

### COVID-19, Diabetes, Mitochondria, VDAC1, and Metformin

The COVID-19 pandemic has been the focus of global concern since its outbreak in December 2019 when a new coronavirus (SARS-CoV-2), was first discovered in Wuhan, China. This virus, that rapidly spread around the world, is characterized by a severe acute respiratory syndrome (Perlman, 2020).

It is identified by the presence of a “crown” structure observed by electron microscope. The whole genome has been sequenced and is composed of a single-stranded RNA about 30Kb in length (GenBank no. MN908947), encoding 9,860 amino acids (Chen L. et al., 2020).

The surface of the COVID-19 virus is covered by a large number of spike glycoproteins that are responsible for binding to the host receptor and membrane fusion (Huang et al., 2020; Letko et al., 2020). To date, most evidence points toward angiotensin-converting enzyme 2 (ACE2) as the primary receptor for virus entry into host cells (Zhao et al., 2020). Genetic polymorphisms of ACE2 are associated with hypertension, cardiovascular disease, stroke, and diabetes (Ranadheera et al., 2018).

Several reports indicate that people with diabetes who become infected by COVID-19 have more severe consequences and a higher risk of mortality compared to non-diabetic individuals (Apicella et al., 2020). Retrospective studies in patients with T2DM hospitalized for COVID-19 suggest that anti-hyperglycemic agent metformin treatment is associated with a threefold decreased risk of death.

A study involving more than 2,500 people with COVID-19 and with T2DM from 16 hospitals in China found an increased incidence of acidosis, although this incidence was not associated with greater mortality in people treated with metformin during hospitalization (Cheng et al., 2020). In addition, metformin was significantly associated with reduced mortality in women with obesity or T2DM who were admitted to hospitals with the virus (Bramante et al., 2021).

Immunomodulatory and antiviral activity of metformin and its potential implications in treating COVID-19 and lung injury has been reported (Chen L. et al., 2020).

It has been hypothesized that ACE2 causes acute lung injury by triggering autophagy through the AMPK/mTOR pathway (Zhang et al., 2019). AMPK increases the expression of ACE2 and its stability by phosphorylating ACE2 Ser^680^ (Liu et al., 2019). Thus, it was proposed that metformin could prevent the entry of SARS-CoV-2, as well as activation of ACE2 through AMPK-signaling (Sharma et al., 2020).

In addition, the SARS-CoV-2 life cycle depends on modulating the mTOR protein and pathway. mTOR signaling is necessary for viral translation, and its interruption inhibits viral growth and replication (Ranadheera et al., 2018). Metformin, an FDA-approved mTOR inhibitor, when administered as an anti-hyperglycemic drug in diabetes patients, was found to simultaneously act as an anti-hyperglycemic and antiviral agent (Lim et al., 2021), offering benefits in patients with COVID-19. This correlation could justify the reduced risk of mortality in metformin-treated compared with non-treated diabetic patients. However, additional studies are necessary to further elucidate the exact role of mTOR inhibitors and modulators in the treatment of COVID-19.

Many viruses modulate mitochondria (Monlun et al., 2017; Tiku et al., 2020). The connection of mitochondria and VDAC1 to the metformin effects on cell function presented above has also been demonstrated for COVID-19 (Thompson et al., 2020). Recently, it was shown that SARS-CoV-2 RNA and proteins are localized to the mitochondria, hijacking the host cell’s mitochondrial function, and manipulating metabolic pathways to their own advantage (Singh et al., 2020; Ajaz et al., 2021).

It was demonstrated that metabolic programs define dysfunctional immune responses in severe COVID-19 patients (Thompson et al., 2020). Moreover, VDAC1 expression level was highly increased in T-cells from these patients, leading to mitochondrial dysfunction and apoptosis (Thompson et al., 2020). In addition, COVID-19 patients’ T-cells underwent apoptosis that was inhibited by VBIT-4 (Thompson et al., 2020), a compound that targets VDAC1 oligomerization and prevents apoptosis (Ben-Hail et al., 2016). Further, VBIT-4 restored insulin secretion in T2DM islets and maintained normal glucose levels and insulin secretion in db/db mice (Zhang E. et al., 2019). Moreover, HK-II was found to be highly expressed in T-cells in acutely ill COVID-19 patients, but not in other viral diseases (Thompson et al., 2020).

These findings point to the likelihood that the mitochondria, VDAC1, and HK are involved in metformin-reduced mortality of T2DM induced by COVID-19.

It should be mentioned that metformin can be considered to be either a friend or foe of SARS-CoV-2-infected patients with diabetes (Ursini et al., 2020).

## Metformin as an Anti-Aging Agent

The nine biological hallmarks of aging include mitochondrial dysfunction, altered intercellular communication, loss of proteostasis, telomere attrition, deregulated nutrient sensing, genomic instability, cellular senescence, stem cell exhaustion, and epigenetic alterations. All have been associated with various neurodegenerative diseases (Hou et al., 2019). Several studies using preclinical models suggest that metformin is improving health span and lifespan (Martin-Montalvo et al., 2013; De Haes et al., 2014; Alfaras et al., 2017; Piskovatska et al., 2019).

In a rat model and human neuronal cell cultures, metformin has been reported to significantly stimulate the formation of new neurons, i.e., neurogenesis, but there has been no sufficient evidence of clinical trials to confirm these findings to date (Potts and Lim, 2012). Mice treated with metformin have been found to live nearly 6% longer than controls, and diabetic patients treated with metformin live 15% longer than healthy individuals without diabetes (Bannister et al., 2014).

Metformin is considered an anti-aging medication as it has been shown to affect many factors that accelerate aging., such as protecting against DNA damage, mitochondrial dysfunction, and chronic inflammation (Formoso et al., 2008; Martin-Montalvo et al., 2013; Cameron et al., 2016; Garg et al., 2017; Valencia et al., 2017). Metformin increases the levels of mTOR and AMPK, which are considered to be longevity-promoting signaling molecules in cells (Martin-Montalvo et al., 2013; De Kreutzenberg et al., 2015). Finally, it has been reported that the AMPK activity declines with age (Salminen et al., 2011). Thus, the finding that metformin activates AMPK may support the suggestion that it is an agent that prevents age-related disorders including cancer, cardiovascular disease, obesity, and neurocognitive decline (Wang et al., 2011; Coughlan et al., 2014; Wang B. Z. et al., 2019).

A decline in mitochondria quality and activity is associated with normal aging and correlated with the development of a wide range of age-related disorders, particularly neurodegenerative diseases. Impaired mitochondrial function includes decreased oxidative phosphorylation (OxPhos), ATP production, mitochondrial dynamics, and mitochondrial quality control, as well as a significant increase in ROS generation, diminished antioxidant defense, and enhanced mitochondria-mediated apoptosis (Chistiakov et al., 2014; Sun et al., 2016). In addition, an accumulation of mutations in mitochondrial DNA (mtDNA) causes adverse effects including altered expression of OxPhos complexes, thereby, decreasing energy production and enhancing ROS generation (Wallace, 2010).

Several mechanisms underlying the anti-aging effect of metformin have been proposed including metformin reducing the production of mitochondrial ROS through the inhibition of complex I (Owen et al., 2000); upregulating ER glutathione peroxidase (Fang et al., 2018); regulating mitochondrial biogenesis and senescence through AMPK-mediated H3K79 methylation (Karnewar et al., 2018); decreasing the opening of the mitochondrial permeability transition pore (mPTP) (Guigas et al., 2004); and inducing autophagy by AMPK activation, regarded as health span-promoting and pro-longevity properties (Piskovatska et al., 2019), and with beneficial effects on chronic inflammation (Saisho, 2015), a state known to contribute to the development and progression of all age-related disorders.

Finally, metformin via binding to SHIP2 prevented Akt inhibition (Polianskyte-Prause et al., 2019) allowing Akt to phosphorylate HK, thereby, increasing its binding to mitochondria (Roberts et al., 2013) and preventing apoptosis.

Several studies demonstrated age-related changes in VDAC isoform expression levels and posttranslational modifications (Groebe et al., 2010). Moreover, an age-dependent increase in VDAC1 in the cerebral cortex of mice has been demonstrated (Manczak and Reddy, 2012).

The increase in VDAC1 expression levels by metformin (Loubiere et al., 2017; Zhang et al., 2017; Duan et al., 2021) can explain its pro-apoptotic effect relevant to cancer therapy. However, no clear mechanism is presented to link between metformin ‘s anti-aging activity and VDAC1. A possible link is metformin inhibiting mPTP opening, and activating mitophagy which removes damaged mitochondria, and is inhibited in aging cells (Rottenberg and Hoek, 2021). VDAC1 has been proposed as one of the components of mPTP (Vianello et al., 2012) and metformin, by blocking VDAC1 conductance (Zhang E. et al., 2019), may inhibit mPTP opening. Thus, metformin’s anti-aging effects may be associated with its effects on mitochondria, HK, and VDAC1 functions. It should be noted that most of metformin’s anti-aging effects were observed at doses that substantially exceed the recommended therapeutic doses in humans (Novelle et al., 2016). Clearly, better understanding of the mechanisms underlying metformin’s effects on health-span and life extension in non-diabetics requires further studies.

## Metformin: Controversial Results and Side Effects

Although metformin used in treating various diseases including diabetes, cancer, obesity, and neurodegenerative and cardiovascular diseases, there are some precautions necessary with its use. Studies on its association with various classifications of age-related cognitive decline have shown diverse results with both positive and negative effects.

The proposed “anti-aging” activity of metformin is a controversial subject in general. The suggestion that it decreases the risk, progression, and severity of AD and other forms of dementia in individuals without diabetes is not supported by the available evidence (Campbell et al., 2018).

Clinical studies have reported that long-term metformin use increased the risk of AD among patients over 65 years old (Imfeld et al., 2012), and T2DM patients treated with it had over two to three times more impaired cognitive function than non-treated patients (Moore et al., 2013). Yet, another cohort study reported that patients with diabetes co-treated with sulfonylureas and metformin alleviated the risk of dementia by up to 35% over an 8-year period (Hsu et al., 2011).

Metformin inhibition of mitochondrial respiration (El-Mir et al., 2000; Wessels et al., 2014) has been shown to contribute to the development of PD. In a cell culture model, it was found to increase Aβ formation (Chen et al., 2009; Picone et al., 2016), and in a population-based study, it increased the rate of AD (Imfeld et al., 2012) and lowered cognitive function in patients with diabetes (Moore et al., 2013).

A recent study demonstrates that metformin increased the generation of Aβ by promoting β- and γ-secretase-mediated cleavage of APP in SH-SY5Y cells. Also, it caused autophagosome accumulation in Tg6799 AD model mice, and it was concluded that it may aggravate AD pathogenesis by promoting amyloidogenic APP processing in autophagosomes (Son et al., 2016). It is proposed that metformin induces Aβ generation by activating AMPK, inhibiting the mTOR pathway, which results in upregulated autophagy and abnormal accumulation of autophagosomes enriched in APP, BACE1, and-secretase, facilitating amyloidogenic Aβ production and AD progression (Son et al., 2016).

In addition, potential side effects of metformin were reported. Typically, gastrointestinal side effects, including diarrhea, nausea, flatulence, indigestion, vomiting, and abdominal discomfort, dominate in individuals taking it (Nasri and Rafieian-Kopaei, 2014). Long-term metformin use resulted in vitamin B12 deficiency (Liu et al., 2014; Niafar et al., 2015), interfering with the absorption of B12 in the terminal ileum (Bauman et al., 2000). Low B12 levels contribute to higher concentrations of artery-clogging homocysteine, an independent risk factor for cardiovascular disease (Ganguly and Alam, 2015). The association between metformin and impaired cognitive function has been linked at least in part to metformin-induced B12 deficiency (Moore et al., 2012; Kim et al., 2019a).

Another side effect is that metformin increases the levels of lactate in mice and humans. Although it is extremely rare, lactic acidosis may cause dizziness, muscle pain, tiredness, difficulty breathing, irregular heartbeat, and stomach pain with diarrhea (Scheen and Paquot, 2013).

## Summary

The interest in metformin has been significantly revitalized during the last years due to its potential repositioning for treatment of many diseases. Metformin has been proposed as a treatment for cancer, and neurodegenerative and other diseases. However, it is not clear what factors mediate its pro-survival or pro-apoptotic activities. Several mechanisms were proposed including activation of the LKB1/AMPK pathway, causing cell cycle arrest, inducing apoptosis, inhibiting protein synthesis and unfolded protein response (UPR), reducing circulating insulin levels, modulating PTP opening, inhibiting mitochondrial complex I, inducing ER stress and increased Ca^2+^ cellular levels, activating the immune system, and more. Here, we propose that metformin interacts with HK, and alters its binding toVDAC1. Together with VDAC1, it regulates cellular energetics and cell death by these proteins. This suggests that metformin’s multiple effects also involve HK and VDAC1, which are both shown to be associated with cancer and neurodegenerative diseases. In cancer, metformin detaches HK from VDAC1, allowing apoptosis, and in neurodegenerative diseases, it interferes with HK phosphorylation and, thereby, allows its bind to VDAC1, protecting against cell death.

## Author Contributions

UA and EN-C contributed to literature search and helped in writing. AS-K contributed in preparing the summary models and the references. MDC helped in the final version of the manuscript. VS-B wrote the manuscript. All authors contributed to the article and approved the submitted version.

## Conflict of Interest

The authors declare that the research was conducted in the absence of any commercial or financial relationships that could be construed as a potential conflict of interest.

## Publisher’s Note

All claims expressed in this article are solely those of the authors and do not necessarily represent those of their affiliated organizations, or those of the publisher, the editors and the reviewers. Any product that may be evaluated in this article, or claim that may be made by its manufacturer, is not guaranteed or endorsed by the publisher.
