# Supplementary material for: Adverse Effects of Metformin From Diabetes to COVID-19, Cancer, Neurodegenerative Diseases, and Aging: Is VDAC1 a Common Target?
Source: Front Physiol. 2021 Oct 4;12:730048. doi: 10.3389/fphys.2021.730048 (PMC8521008; doi:10.3389/fphys.2021.730048)
Supplement: Supplementary file 1 [file Table_1.DOCX]

**Table 1. VDAC1 overexpression is a common factor in metformin affecting diseases**

| **Diseases** | **VDAC1 state** | **Function** | **Refs.** | **Metformin association** | **Refs.** |
| --- | --- | --- | --- | --- | --- |
| Type 2 diabetes (T2DM) | Overexpressed | Impairs generation  of cellular ATP and induced apoptosis | Ahmed et al., 2010;Gong et al., 2012;Sasaki et al., 2012;Zhang et al., 2019a | Improves glucose tolerance | Maruthur et al., 2016;Palmer et al.,2016;Sanchez-Rangel and Inzucchi, 2017 |
| Cancer | Overexpressed | Increases cancer cell metabolic activity | Abu-Hamad et al., 2006;Koren et al., 2010;Arif et al., 2014;Shoshan-Barmatz et al., 2015;Arif et al., 2017;Shoshan-Barmatz et al., 2017;Pittala et al., 2018 | Anti-cancer activity | Chen et al., 2017;Andrzejewski et al., 2018;Biondani and Peyron, 2018;Xie et al.,  2020 |
| Alzheimer’s disease (AD) | Overexpressed | Neuronal cell death | Perez-Gracia et al., 2008;Cuadrado-Tejedor et al., 2011;Manczak and Reddy, 2012 | Neuroprotective | Qiu and Folstein, 2006;Hsu et al., 2011;Rotermund et al., 2018 |
| Parkinson’s Disease  (PD) | Interaction with alpha-synuclein | Regulates VDAC1 conductance and VDAC1-mediated Ca^2+^ transport | Rostovtseva et al., 2015;Rosencrans et al., 2021 | Reverses certain PD phenotypes | Bayliss et al., 2016;Lu et al., 2016;Ryu et al., 2018) |
| Epilepsy | Increased expression | Apoptosis, alerts energy charge | Jiang et al., 2007 | Decreases seizure frequency and duration, stops seizures | Zhao et al., 2014;Yang et al., 2017;H et al., 2019 |
| Depression/  bipolar | Upregulation of VDAC and TSPO | TSPO-VDAC complex down-regulates mitophagic proteins and NLRP3 inflammasome activation | Nahon et al., 2005;Scaini et al., 2019 | Anti-depressant | Guo et al., 2014 |
| Cardiovascular diseases (CVDs) | Overexpressed | Cardiomyocyte cell death | (Lim et al., 2001;Schwertz et al., 2007;Liao et al., 2015;Tong et al., 2017;Jiang et al., 2018;Tian et al., 2019;Yang et al., 2019;Klapper-Goldstein et al., 2020) | Reduces risk of CVDs among patients with T2DM | (Norwood et al., 2013;Griffin et al., 2017;Rena and Lang, 2018;Mohan et al., 2019) |
| Non-alcoholic fatty liver disease (NAFLD) | Overexpressed | Mediates transport of fatty acids across the OMM | (Lee et al., 2011;Tonazzi et al., 2015;Pittala et al., 2019) | Attenuates the onset of NAFLD | (Koren et al., 2010;Brandt et al., 2019) |
| Inflammatory bowel disease (IBD) and gut microbiota composition | Overexpressed | Mediates apoptosis, and inflammation | (Verma et al., 2021) | Affects IBD and intestinal microbiota and is a barrier in small intestine | (Brandt et al., 2019;Ouyang et al., 2020;Tseng, 2021) |
| Rheumatoid arthritis  (RA) | Increased VDAC1 oligomerization | Induces cardiac cell death and functional impairment in RA | (Zeng et al., 2018) | Improves the pathogenesis of RA | (Matsuoka et al., 2020) |
| Covid-19 | Overexpressed | Induction of apoptosis | (Thompson et al., 2020) | Decreases risk of death in T2DM affected by COVID-19 | (Chen et al., 2020b;Luo et al., 2020;Scheen, 2020;Bramante et al., 2021) |

**Table 2. Metformin decreases cancer cell resistance to chemotherapy**

| **Disease** | **Metformin effect** | **Ref.** |
| --- | --- | --- |
| ALL | In patients with higher ABCB1 gene expression levels, the combined use of metformin with chemotherapy is beneficial. | (Ramos-Penafiel et al., 2018) |
| Breast cancer | Metformin reduces the expression of MDR protein markers, prevents the growth of treatment-resistant breast cancer, and fosters re-sensitization. | (Davies et al., 2017) |
| Breast cancer | Metformin re-sensitized multidrug-resistant breast cancer cells (MCF7/5-FU and MDA-MB-231) to 5-fluorouracil (5-FU), adriamycin, and paclitaxel reduced their invasive potential, and reversed the epithelial-mesenchymal transition (EMT) phenotype. | (Qu et al., 2014) |
| Nasopharyngeal carcinoma (NPC) | Metformin reduced the expression of PECAM-1, which controls the expression of the multi-drug expression of resistance-associated proteins (MRPs) that contribute to cisplatin resistance of irradiated CNE-1 cells. | (Sun et al., 2020) |
| Breast cancer | In breast cancer and MCF7/DOX cells, metformin lowers Pgp activity | (Shafiei-Irannejad et al., 2018) |
| Triple negative breast cancer (TNBC) | Metformin increases cisplatin's anti-proliferative, anti-migratory, and anti-invasion effects in TNBC cells.  Metformin also reduces the upregulation of RAD51 expression by triggering RAD51 proteasomal degradation. | (Lee et al., 2019b) |
