# Supplementary material for: Adverse Effects of Metformin From Diabetes to COVID-19, Cancer, Neurodegenerative Diseases, and Aging: Is VDAC1 a Common Target?
Source: Front Physiol. 2021 Oct 4;12:730048. doi: 10.3389/fphys.2021.730048 (PMC8521008; doi:10.3389/fphys.2021.730048)
Supplement: Supplementary file 2 [file Table_2.DOCX]

**Table 2. Metformin decreases cancer cell resistance to chemotherapy**

| **Disease** | **Metformin effect** | **Ref.** |
| --- | --- | --- |
| ALL | In patients with higher ABCB1 gene expression levels, the combined use of metformin with chemotherapy is beneficial. | (Ramos-Penafiel et al., 2018) |
| Breast cancer | Metformin reduces the expression of MDR protein markers, prevents the growth of treatment-resistant breast cancer, and fosters re-sensitization. | (Davies et al., 2017) |
| Breast cancer | Metformin re-sensitized multidrug-resistant breast cancer cells (MCF7/5-FU and MDA-MB-231) to 5-fluorouracil (5-FU), adriamycin, and paclitaxel reduced their invasive potential and reversed the epithelial-mesenchymal transition (EMT) phenotype. | (Qu et al., 2014) |
| Nasopharyngeal carcinoma (NPC) | Metformin reduced the expression of PECAM-1, which controls the expression of the multi-drug expression of resistance-associated proteins (MRPs) that contribute to cisplatin resistance of irradiated CNE-1 cells. | (Sun et al., 2020) |
| Breast cancer | In breast cancer and MCF7/DOX cells, metformin lowers Pgp activity. | (Shafiei-Irannejad et al., 2018) |
| Triple negative breast cancer (TNBC) | Metformin increases cisplatin's anti-proliferative, anti-migratory, and anti-invasion effects in TNBC cells.  Metformin also reduces the upregulation of RAD51 expression by triggering RAD51 proteasomal degradation. | (Lee et al., 2019b) |
